# Public involvement and health research system governance: a qualitative study

**DOI:** 10.1186/s12961-018-0361-6

**Published:** 2018-08-30

**Authors:** Fiona Alice Miller, Sarah J. Patton, Mark Dobrow, Deborah A. Marshall, Whitney Berta

**Affiliations:** 1 Institute of Health Policy, Management and Evaluation, University of Toronto, 155 College Street, 4th Floor, Toronto, ON M5T 3M6 Canada; 2 Department of Community Health Sciences, University of Calgary, 3280 Hospital Drive NW, 3rd Floor, Calgary, AB T2N 4Z6 Canada

**Keywords:** Public involvement, community-based research, health research systems, research governance, research policy

## Abstract

**Background:**

Interest in public involvement in health research projects has led to increased attention on the coordination of public involvement through research organisations, networks and whole systems. We draw on previous work using the ‘health research system’ framework to explore organisational actors and stewardship functions relevant to governance for public involvement.

**Methods:**

To inform efforts in Ontario, Canada, to mobilise public involvement across the provincial health research enterprise, we conducted an exploratory, qualitative descriptive study of efforts in two jurisdictions (England, United Kingdom, and Alberta, Canada) where there were active policy efforts to support public involvement, alongside jurisdiction-wide efforts to mobilise health research. Focusing on the efforts of public sector organisations with responsibility for funding health research, enabling public involvement, and using research results, we conducted in-depth, semi-structured interviews with 26 expert informants and used a qualitative thematic approach to explore how the involvement of publics in health research has been embedded and supported.

**Results:**

We identified three sets of common issues in efforts to advance public involvement. First, the initial aim to embed public involvement leveraged efforts to build self-conscious research ‘systems’, and mobilised policy guidance, direction, investment and infrastructure. Second, efforts to sustain public involvement aimed to deepen involvement activity and tackle diversity limitations, while managing the challenges of influencing research priorities and forging common purpose on the evaluation of public involvement. Finally, public involvement was itself an influential force, with the potential to reinforce – or complicate – the ties that link actors within research systems, and to support – or constrain – the research system’s capacity to serve and strengthen health systems.

**Conclusions:**

Despite differences in the two jurisdictions analysed and in the organisation of public involvement within them, the supporters and stewards of public involvement sought to leverage research systems to advance public involvement, anticipated similar opportunities for improvement in involvement processes and identified similar challenges for future involvement activities. This suggests the value of a health research system framework in governance for public involvement, and the importance of public involvement for the success of health research systems and the health systems they aim to serve.

## Background

Public involvement in health research is understood as research that is “*carried out ‘with’ or ‘by’ members of the public rather than ‘to’, ‘about’ or ‘for’ them*” [1]. Much public involvement is pursued within individual research projects or programmes of research where publics advise on research design or conduct, or participate as investigators in data collection or analysis. Additionally, public involvement is relevant to the activities of the organisations that fund or otherwise guide or support research, through advising on research priorities, applications or processes [1, 2]. Driven by both instrumental and deontological aims, public involvement is seen as a way for the conduct and outcomes of research to serve the interests, and be informed by the lived experience, of the persons affected by illness and the products, services and systems used to address it (patients, families, informal caregivers), the communities who collectively encounter the social, economic and political circumstances that condition health outcomes and opportunities for their redress, and the lay publics who finance and give warrant to the research enterprise [2–6].

Growing interest in public involvement in health research has led to substantial scholarly and policy effort. Alongside a burgeoning literature that documents the processes and outcomes of public involvement, typically with a focus on individual research projects or programmes [7–10], growing efforts by governments and other public sector agents to enable, coordinate and evaluate such activity has been matched by emergent scholarly interest in the nature and effects of such organisational and whole-system change [11–14].

Policy-oriented scholarship on public involvement has begun to document the nature of the policy activity, the types of organisational and network actors, and the social and political tensions that condition the realisation of public involvement across the health research enterprise [15]. Such research illustrates the important role of governments and public policy in responding to and enabling the public involvement agenda [6, 16]. Additionally, it highlights the important role of the policy and practice of research producer organisations in absorbing and enacting this commitment [3, 4]. There is also particular attention to research funding agencies [17–19], which, as Van Bekkum et al. [20] have argued, play “*an active role in shaping the boundaries of PE* [public engagement] *within their research communities*”. Further, funding agencies may forge network connections with scientists and other organisations in ways that constrain public involvement, with the existence of what Caron-Flinterman et al. [2] have characterised as “*regimes*” of “*dominant cultures, stabilized patterns of interaction, usual practices and established institutions*”, in which publics are necessarily marginal players.

Tacit or explicit in these explorations are expectations for what the public involvement agenda will yield – expectations that are not necessarily shared by all research system stakeholders [21, 22]. Indeed, as Green has recently highlighted, there are persistent tensions between a consumerist and more democratic or emancipatory paradigm for public involvement – the former seeking to “*improve the product*” though better utilisation of relevant expertise, the latter seeking to provide publics with “*more say in agencies, organisations, and institutions which impact upon them and* [the ability] *to exert more control over their own lives*” [23]. Green documents the many successes of the public involvement agenda in the United Kingdom through policy and infrastructure development, as well as the instantiation of public involvement activity at strategic levels and within funding allocation processes, but also wonders at the limited extent to which public involvement has led to a “*transformation of the social relations of research, as envisaged by the emancipatory research movement*” [23].

These developments and debates raise questions of ‘governance’, that is, the way rules, norms and actions are structured, sustained and regulated by public and para-public actors to condition the operation and impact of public involvement activities [24, 25]. In previous work, we explored the value of a ‘health research system’ [26] approach to governance for public involvement, and suggested its relevance for efforts to embed public involvement system-wide, and in specifying the role for publics in adjudicating and legitimising health research systems [15]. Additionally, this work highlighted the particular importance of organisational actors, such as funding agencies, universities or research hospitals, in advancing public involvement across inter-organisational networks and whole systems, and the particular significance of what Pang et al. [26] have characterised as the stewardship function in health research systems, which sets the overall direction for the research enterprise, balancing demands among and across the system’s many stakeholders [21, 22].

Thus, to inform efforts in Ontario, Canada, to advance public involvement across the provincial health research enterprise, we explored how two jurisdictions have worked to embed and support public involvement in health research, with a specific focus on the stewardship function and the role of key organisational actors that enable, support and also delimit public involvement activity.

## Methods

We conducted a qualitative descriptive study from January to April 2017 to explore how the involvement of publics in health research is governed, including how public involvement is embedded and supported by public and para-public actors. Our aim was to examine jurisdictions where there was an active policy effort to support public involvement, alongside jurisdiction-wide effort to mobilise health research. We selected England because it is widely seen as exemplary for the duration and depth of effort to mobilise and diffuse public involvement in health research [1, 6, 16, 20, 23, 27], and Alberta because it met our criteria and our desire to explore public involvement efforts in a Canadian context to inform parallel efforts in Ontario.

We focused our attention on organised effort within each jurisdiction, adapting Caron-Flinterman et al.’s [2] concept of the intermediary organisation. Specifically, we recruited informants from organisations with a core and broad role in research systems, such as funding agencies, as well as organisations with a specific role in enabling public involvement within such systems, namely organisations that exist to support public involvement through training, linkage or strategic leadership. For Caron-Flinterman et al. [2], intermediary organisations include those in the public and private sectors, and their description suggests that organisations that demand, utilise and often partner in the production of health research are not ‘intermediaries’ in the sense intended. Given our aim to inform governance of and by public sector organisations, and the need to maintain a manageable scope, we focused on public sector organisations. Additionally, given our interest in research mobilisation and the relevance of public involvement for research use, we also engaged key public sector organisations that ‘use’ health research such as health service agencies and government departments or Ministries of Health.

The political intent and terminology related to ‘public involvement’ is complex and contested. In the United Kingdom, public involvement and public engagement are distinct concepts, with public involvement representing higher levels of participation and control than public engagement [28]. In Canada, the meaning of the terms is reversed, with engagement often implying a higher level of participation and control than involvement [29]. Further, many ‘publics’ might be involved and the terms used to describe each vary. We approached respondents using the language of public engagement or involvement, which we did not define, but noted our understanding of the relevance of this concept for three overlapping constituencies, namely patients (service users, clients, consumers, families, informal caregivers), collective communities (defined by common history, socioeconomic status or ethnocultural identity), and lay publics. We asked informants to use the terms that they preferred but we use ‘involvement’ and ‘publics’ here as generic terms, unless a different concept was clearly intended.

In-depth, semi-structured interviews were conducted with 26 key informants, including 10 from England and 16 from Alberta. We recruited informants with expert knowledge of organisational and jurisdictional effort to support public involvement in health research, sampling across three types of ‘intermediary’ organisation, as follows: (1) public sector health research funding agencies; (2) organisations with a dedicated role in supporting public involvement in health research; and (3) organisations that are key publicly funded users of health research (which may also produce or co-produce health research). In addition, to gather insights from informed observers of public involvement effort within each jurisdiction, (4) we interviewed academic researchers with established expertise in public involvement in health research (Table 1).

**Table 1:** Type and total number of informants interviewed

Type of informant	England (*n*)	Alberta (*n*)
(1) Public sector health research funding agencies	2	4
(2) Organisations with a dedicated role in supporting public involvement in health research	5	4
(3) Organisations that are key publicly-funded users of health research	1	7
(4) Academic researchers with expertise in public involvement in health research	2	1
Total interviewed: (*n* = 26)	10	16

Informants were recruited through personal contacts or direct outreach. Interviews were conducted by telephone at times of mutual convenience. Written consent was obtained from respondents for participation in interviews. Research ethics approval was obtained from the University of Toronto Health Sciences Research Ethics Board.

We used a semi-structured but open-ended approach to interviews, to permit informants to interpret and answer questions as they saw fit, and allow unanticipated ideas and themes to emerge [30]. The basic thematic structure of the guide was informed by the four operational components of the stewardship function identified by Pang et al. [26]. Specifically, we asked informants about the vision for public involvement in their organisation or jurisdiction, how public involvement activity was coordinated, public involvement in research priority-setting and standard setting for public involvement, as well as monitoring and evaluation of public involvement. This basic thematic structure remained common across all interviews, with modifications and additions as new themes and issues arose over the course of the interviews [30].

### Analytic approach

All interviews were recorded, transcribed verbatim and coded. Data analysis began in the early stages of data collection and was informed by ongoing interviews. We drew on the interview guide themes as organising concepts for initial descriptive analyses of our data. Then, drawing on the principles of constructivist grounded theory, we modified and reorganised codes and identified new and cross-cutting themes through an iterative process using constant comparison, whilst using memos throughout [31–33]. We member checked a penultimate version of our findings with informants, seeking feedback for clarification and comments on our interpretation.

Although we analysed public involvement within two jurisdictions, our aim was not to comprehensively compare and contrast these as distinct cases. Rather, we sought to collect and analyse data to identify processes and challenges that were consistently present – if differently addressed – across jurisdictions. This aligned with our aim, which was to identify opportunities for advancing public involvement in Ontario based on the experiences and expectations of leaders and observers of such efforts in other jurisdictions.

### Introduction to two health research systems

Our study reports on public involvement in two jurisdictions with important similarities and differences (Table 2). A notable difference between the two jurisdictions is that of size. England is the largest of four countries in the United Kingdom, with a population of 55.3 million [34]. By contrast, Alberta is the fourth largest province in Canada, with a population of only 4.3 million [35]. Yet, both jurisdictions share some of the complexities associated with federal or quasi-federal states. In the United Kingdom, both healthcare and higher education – where the bulk of public sector health research is conducted – are devolved responsibilities. Thus, there is England-specific legislation and policy for the English National Health Service (NHS), and for the country’s universities. By constitution, Canada is a decentralised federation. Thus, both Alberta Health Services and Alberta’s universities are organised and overseen by the provincial legislature, though the federal government provides financial support to healthcare under a set of basic conditions that create core commonalities across Canada’s provincial and territorial health systems.

**Table 2:**
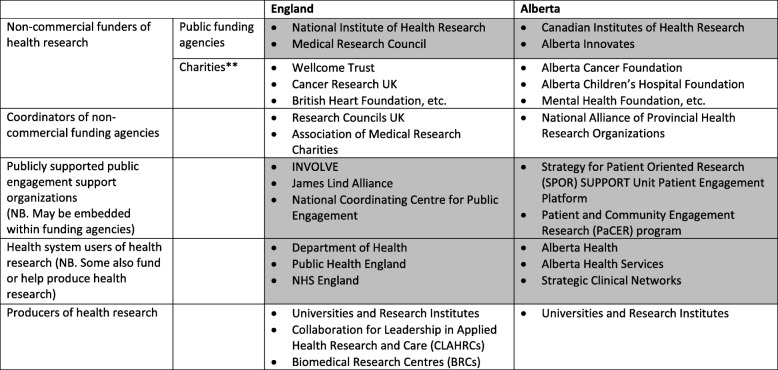
Public engagement in health research systems – key public sector ‘intermediary organisations’^a^

^a^Shaded areas identify the types of organisations from which key informants were recruited

**We include charities for completeness in identifying non-commercial funders, though these are third sector rather than public sector organisations

The size differences between the two jurisdictions manifest with respect to health research capacity, though similarities are also present with respect to the distribution of responsibilities in health research funding. England houses many funding agencies (public, third sector and private) that support health research. Key public-sector funding agencies include the National Institute for Health Research (NIHR), which funds applied health research, serving as the ‘health research system’ for NHS England. The Medical Research Council funds basic health research, and unlike NIHR, funds research across the United Kingdom. Both the Association of Medical Research Charities and Research Councils UK provide some coordinative capacity for United Kingdom research, though the remit of Research Councils UK extends far beyond health research. Key public sector users of health research in England include NHS England and Public Health England.

In Alberta, the provincial funding agency, Alberta Innovates, supports a broad portfolio of research, with a dedicated health research portfolio named Health Solutions. The Canadian Institutes of Health Research (CIHR) is the federal health research funding agency that supports a full spectrum of health research across Canada. Both CIHR and the National Alliance of Provincial Health Research Organizations, which represents provincial health research funding agencies, provide some coordinative capacity for health research across the country. In Alberta, key public sector users of health research include the Department of Health, Alberta Health Services and the Strategic Clinical Networks, a novel organisational form that mobilises research in service of high quality comprehensive clinical care pathways, across and beyond the Alberta Health Services.

Both jurisdictions house dedicated infrastructure to support public involvement in health research. In England, sustained effort since the 1990s by governments and health research funding agencies has advanced public involvement in health research. The NIHR, in particular, has a longstanding commitment to public involvement, dating from its precursor organisation, NHS Research and Development [36, 37]. It has supported the creation and maintenance of substantial public involvement infrastructure, notably INVOLVE, one of the more established public involvement support organisations in the world, and also funds and houses the James Lind Alliance, a centre to support public and clinical involvement in research priority-setting [23]. The Medical Research Council also made a commitment to public involvement with the creation of the Consumer Liaison Group, renamed the Advisory Group for Public Involvement, dating from the 1990s [27]. The National Coordinating Centre for Public Engagement also emerged in the 2000s to support English universities in their public involvement and engagement efforts, including and beyond health research.

In Alberta, the Strategy for Patient Oriented Research (SPOR) of the CIHR has encouraged the growth and codification of public sector commitment to public involvement [38–40]. This has led to the creation of a public involvement platform within Alberta Innovates. Additionally, and in part responding to these initiatives, another key public involvement support infrastructure has developed in Alberta – the Patient and Community Engagement Research (PaCER) initiative – which trains and supports members of the public to conduct research, often in close collaboration with the Strategic Clinical Networks [41].

## Results

As noted above, the aim of our analysis was not to characterise public involvement in each research system, but to identify issues that were consistent across and related to both organising and embedding public involvement. Specifically, we identified a common set of issues and challenges related to efforts to support and sustain public involvement in health research, arising from (1) the initial aim to embed public involvement in health research, mobilising what we characterise as key building blocks, (2) the ongoing need to sustain public involvement by enriching and evaluating it, and (3) the implications of such efforts for health research and its health system users. We present our findings across these three ‘stages’ of effort and reflexive consideration, identifying relevant sub-themes within each.

### Part 1. Embedding public involvement: building blocks

In both jurisdictions, we identified a similar set of basic building blocks that were seen to have enabled public involvement to be embedded within and across health research organisations and practices. A first building block was a self-aware health research system – one that exists or aims to exist. A second building block leveraged this capacity, embedding public involvement in and across organisations and practices through committed leadership, supportive policy, and enabling infrastructure and resources.

#### A health research system aims to exist

With very few exceptions, respondents from England and Alberta recognised and endorsed the concept of a health research system, seeing the idea as relevant to their efforts. In a more or less developed form, a health research system was seen to exist in both jurisdictions, specifically one that aimed to knit together the individuals and organisations with a role in mobilising, producing or using health research.

In England, the system was present in “*the attitudes of the people working within it and the structures set in place …*.” (E5). Further, this ‘system’ had been actively created:

“*Yes, I think everything in England over the last 10 years is about … creating a very connected health research system that is highly collaborative, highly effective and efficient.*” (E1)

In Alberta, a health research system was also seen as something both present and actively worked upon: “*Oh I think it’s present in Alberta; that’s what we are currently working on …*” (A1). This system extended beyond any one organisation, being manifest in a network of interconnected actors, including the provincial health service, academic organisations and health charities.

Though typically endorsing its existence in principle, many respondents saw room for continued improvement in the nature and workings of the health research system. There was a continued need to build linkages to ensure the system’s effective operation. As one informant from England noted, day-to-day operations did not necessarily “*feel like a working system*” (E6). Similarly, an informant from Alberta noted that the health research system still lacked the coordination and exchange dynamics that characterise a true ‘ecosystem’:

“*So we talk about the research and innovation system here in Alberta, but the reality is though it’s not really a high performing system and … The parts are not all interconnected, they’re not all linked, all aligned, right? So it’s not really a system … we talk about it; Canada talks about it like it is the ecosystem, but people from the environmental sector tell me that a real ecosystem relates and coordinates. So we talk about that a lot but we don’t do a lot …*” (A7)

#### Factors for embedding public involvement in the health research system

Across both England and Alberta, efforts have been made to embed public involvement within the respective health research systems. Informants identified several ways in which this embedding has been pursued, including the commitment of senior leadership, the mobilisation of supportive policy, the creation and use of supportive infrastructure, and the availability of human and financial resources.

Many informants were quick to identify the importance of the commitment of senior leadership in moving the public involvement agenda forward within each jurisdiction. In the United Kingdom, a former Chief Medical Officer’s vision that public involvement be built into the work of the NIHR was often cited as essential to the agenda’s success:

“*We had a Chief Medical Officer …* [who] *was very instrumental in setting up the NIHR and they had a very clear vision about public involvement in that and so she very much championed that, which was huge for us here.*” (E5)

In Alberta, informants also identified visionary leadership as a key factor in the more recent embedding of public involvement within the health research system.

Yet, the leadership of key individuals did not only manifest through visionary statements; it was also made tangible through policy and, in some cases, strict requirements. As one informant from England noted, the important role that the Chief Medical Officer had played involved imposing public involvement as a requirement for health research funding, saying that “*you’re either going to do this or I’m not going to give you any money*.” As the informant went on to note:

“*So I think if you are going to create a vision … And if you are going to state it’s got to be a core part of what the system looks like then you also have to make sure, then it has to be sort of written in stone, the leaders of that will live it and preach it.*” (E1)

Many informants highlighted how broader forces such as government reports and associated policies influenced the evolution of public involvement in health research within their jurisdiction. In England, the Department of Health’s Best Research for Best Health (2006) and the NIHR’s Going the Extra Mile (2015) were seen as having played a key role in the evolution and development of a vision for public involvement. Similarly, in Alberta, informants often cited Alberta Health Services’ Patient First Strategy (2015) and CIHR’s SPOR (2011) as critical to their success.

In addition to leadership and supportive policy, public involvement was embedded within health research systems through the mobilisation of organisational infrastructure and the associated human and financial resources. In England, for example, several informants pointed to the importance of INVOLVE as a national advisory group to support public involvement in health research, which worked both formally and through advocacy to advance public involvement efforts.

In Alberta, informants also highlighted the importance of new infrastructure devoted to public involvement as well as the existence of health system infrastructure that was supportive of the public involvement agenda. A key piece of new infrastructure in Alberta was the SPOR SUPPORT Unit, which included a dedicated patient involvement platform. For several informants, this dedicated and distinct platform existed “*to make sure the focus of patient-oriented research was not tokenistic but rather seriously considered*” (A1).

Such infrastructure embodied and enabled the commitment of human and financial resources to public involvement efforts. Personnel with expertise in public involvement were seen as critical to the coordination and support of involvement efforts. In Alberta, a key element in the development of public involvement capacity was a training programme to enable patients to participate actively as researchers. In England, varied training initiatives exist to support public involvement, with a similar commitment to a “*skilled workforce around public involvement*” (E3).

Informants with whom we spoke had variable insight into the funding arrangements that supported public involvement capacity within their organisation or jurisdiction. Thus, only some commented on the adequacy of available funds. Many, however, shared a common concern about issues of long-term financial security for public involvement efforts given the financial constraints within healthcare systems. As one informant from England noted, there was always the risk that public involvement could be seen as nice-to-have rather than a necessity in times of austerity:

“*I think it’s a challenging environment … I think it’s one of those things that you know, in a time of budget cuts and austerity, people very easily put a red line through it, you know, just, well, we don’t need that. I think you’re constantly fighting that temptation, that sort of trigger pull that people do when cutting budgets or reducing funds … so it’s a constant battle.*” (E1)

### Part 2. Sustaining public involvement: enriching and evaluating

Anxieties about the future did not detract from a general sense that efforts within each jurisdiction to advance public involvement in health research had been successful. Informants reflected, therefore, on the fact of their success and on the next steps required for the enrichment and deepening of public involvement. Additionally, they reflected on the way in which the ‘success’ of public involvement in health research systems might be monitored and evaluated.

#### Changing culture, changing practice – the long road to public involvement

There was a strong sense that the aim to embed public involvement within the health research enterprise within each jurisdiction had been quite successful. At the same time, informants identified opportunities to further enrich the involvement mandate, addressing the challenge of diversity among persons involved, and of involving publics in setting priorities for health research.

Though acknowledging continued resistance among some researchers, many informants identified a broad ‘cultural shift’ in favour of public involvement in health research. As one informant from England put it:

“*I think there has been this general cultural sense that the public citizen should be more involved in all services, in health services particularly and even more particularly in health research.*” (E4)

As public involvement had become better accepted across the health research enterprise, there was increased opportunity to further expand and refine involvement efforts. In part, this related to the vision for involvement, which had initially been quite simple. As one informant from England noted, the initial aim had simply been to “*involve the public in every aspect of the research cycle*”. With time, however, the mandate became more sophisticated: “*the questions we’re now posing … are different, or bigger in nature*” (E1). For this informant, research organisations were at “*a crossroads*” in public involvement, with an opportunity to adopt a more intentional approach, where involvement efforts are led by purpose:

“*So I think there’s probably, we’re probably at a crossroads in some respect in public involvement in that we need to move from a fairly dogmatic, fairly blind approach to a much more pragmatic one in which our involvement work is really led by purpose. You know, what’s the purpose of doing this; is public involvement the right thing and if it is the right thing, who then needs to be involved in this. At the moment, there’s a little bit of a system default going on which is, we need to do public involvement, let’s get anybody in that we can possibly think of because we just need to tick a box.*” (E1)

For many informants, efforts to enrich public involvement and be more purposeful in its execution required addressing issues of equity and inclusion, given that the person most often engaged is “*white, educated, middle class and retired*” (E2). This might require public involvement efforts organised to address community needs or addressing some of the factors that limit involvement. As one informant from England noted, the lack of diversity among involved publics reflected structural challenges, thus discouraging participation of many of “*the people who should get involved*”:

“*The people who actually get involved are different probably than the people who should get involved … So there are a whole range of structural reasons. So the whole issue around payment, you know, the whole issues around benefits, welfare benefits and tax. So that in itself is a massive issue around equity. The people who probably should be involved may be not be because if they, for example, were to be paid then they jeopardise their welfare benefits …*” (E6)

Another public involvement issue that demonstrated both successes and challenges related to the involvement of publics in setting priorities for research – an issue we probed, given its central importance in the stewardship of health research systems. In both jurisdictions, there was attention to the importance of involving publics in research priority-setting, though coordinated processes for doing so differed. Informants from England pointed to the well-established James Lind Alliance, as well as other priority-setting processes within NIHR and its regional branches. In Alberta, there was less consensus about the existence of processes to foster public involvement in research priority-setting, though some informants pointed to various processes, including through Alberta’s Strategic Clinical Networks. Despite these differences, there were important similarities in reflections on both the importance and challenge of involving publics in these processes. A common concern was the sense of a ‘mismatch’ between the aims of researchers and the aims of publics with respect to research priorities. As an informant from England put it:

“*So then, you know, there isn’t necessarily a direct match between things that members of the public want to be researched and things that researchers want to research themselves.*” (E3)

Public involvement in research priority-setting faced persistent challenges. Members of the public were not always well equipped to identify their needs, or characterise these as researchable questions. Researchers, by contrast, were consistently clear in their ability to advance their ideas. As a result, as one informant from England put it, “*I think there’s just so many agendas in research priority-setting that the public tend to get squeezed out*” (E4). Additionally, processes to analyse and act upon priorities identified by publics were seen as lacking, even where exemplary processes existed to identify them. Highlighting the challenge of ‘mismatch’ as an issue of power and resistance by researchers, who doubted the “*added incremental value of asking patients what we should be working on for research*”, another informant from Alberta suggested that this was one of the biggest challenges facing their involvement efforts:

“*I think one of the biggest challenges we’re having is probably the value proposition with the research community, which has been pretty hard-nosed about the added incremental value of asking patients what we should be working on for research. We already know their issues … we’ve been in the field for 30 years. I mean, I know what patients need. And so that power balance is a bit of a struggle.*” (A7)

#### Evaluating public involvement in the health research system

The sense of growth and growing success of public involvement in health research was accompanied by increased attention to the issue of evaluating such public involvement efforts. We probed informants about a range of issues related to the evaluation of public involvement within health research systems, including the existence of evaluation frameworks or efforts, and the development of standards or benchmarks to support the assessment of performance. As our informants made clear, this was an area of developmental effort across our two jurisdictions, though it was not without some uncertainty and tension.

Many informants advanced arguments in favour of the evaluation of public involvement across health research systems, but offered two rather distinct sets of ideas. On the one hand, efforts to monitor or evaluate public involvement were understood as fundamentally supportive of the enterprise, by aiming to improve and advance a still-developing science and practice. On the other hand, efforts to monitor or evaluate public involvement were understood as more frankly ‘evaluative’ in nature, seeking to use monitoring and performance assessment to justify the enterprise by demonstrating that it worked.

For many informants, monitoring and evaluation of public involvement was needed to make it better. Public involvement was, some informants suggested, a new ‘science’ to be improved upon. As one informant from Alberta put it, evaluation is “*critical to everything we do because the science of patient engagement is weak and we want to come out of this with some best practices …*” (A6). Evaluation was therefore needed to know “*what works and what doesn’t work and where we’re making progress*” (A10). In a similarly supportive vein, some informants perceived monitoring and evaluation as a way to advance the practice of involvement by helping to make the process clear and tractable, where evaluation “*turns something too woolly and abstract into something very practical. If you want change to happen you need to be very practical*” (E9).

For other informants, monitoring and evaluation of public involvement was more clearly connected to its justification to external stakeholders. Pointing frequently to accountability expectations from public funders, and the fact of budgetary constraints, these informants highlighted the need to demonstrate that “*it’s value-added for everybody involved*” (A4). Similarly, this informant from England noted the importance of evaluation for a publicly funded organisation supporting public involvement:

“*And I think particularly if you are a public funder spending public money with a public face, I think you have to be prepared to answer the question really, really well.*” (E1)

These two views of evaluation were not necessarily contradictory. Indeed, some informants were explicit in seeing evaluation and monitoring of public involvement as a way to both improve upon, and prove the value of, involvement. As one informant from England put it, evaluation initiatives that advanced and assessed public involvement were part of advancing the now-accepted practice:

“*Yeah, I mean I think we’ve got now, okay, patient and public involvement is happening … we’ve got to that stage and now you know everybody accepts it as normal practice. So now we’re kind of at the next steps. How do we refine it further? … So now, the question is, how can we do this more efficiently or how can we, you know, prove what we do is meaningful and also look to see where our weaknesses are and what we can do to improve on those areas.*” (E2)

Yet, these two views of the purpose of evaluation also aligned with a more critical view of the evaluative enterprise. As this informant went on to explain, there is debate regarding the evaluation of public involvement, with some viewing it as essential and others considering it inappropriate:

“*So, well, that’s a bit of another area of debate. This is another area that had two very strong camps that can be opposing views. It is very interesting. You know, some are very adamant that you know, patient and public involvement is something that can and must be measured and another camp of people who think you know it is a moral right and you don’t go around measuring that but rather you must ensure that the processes are there to enable the public to be involved…*” (E2)

Several informants elaborated these concerns, which were particularly aimed at the use of evaluation to assess or justify the enterprise. These informants suggested that expectations of evaluative performance were both unrealistic and inappropriate. As one informant noted:

“*I mean I’m all for evaluation but sometimes it feels like since we involve patients, we gotta all of a sudden evaluate it but if we just do it ourselves, they just leave us alone, right? Like there’s something profoundly disturbing about that…*” (A17)

In a similar vein, another informant characterised such evaluative efforts as ‘reductionist’ and unsuitable to the exercise of public involvement:

“*There’s a slightly reductionist approach to evaluating PPI* [patient and public involvement] *– you know, can you demonstrate through CTs* [clinical trials] *that public involvement leads to good quality research? Well actually, no, it’s very difficult to do that and so you know, we don’t ask health economists and statisticians to justify their, you know, their space in the research world through CTs but so I just think we’re really asking the public voice to be evaluated in a way that is very difficult to do … So I think that is, that’s quite a danger actually.*” (E4)

### Part 3. Implications of public involvement: health research systems and health systems

Though not without challenges and uncertainties, public involvement in health research was seen as substantially embedded within the two health research systems we studied. To accomplish this, initiatives to advance public involvement had leveraged existing ‘system’ capacity. In turn, such public involvement initiatives had the potential to affect the aims and operations of these same systems. Thus, informants reflected on the ways in which public involvement may affect the health research system with respect to its internal functioning and its orientation toward the health system it aims to serve.

#### Implications of public involvement for health research systems

Systemic efforts to embed public involvement within the health research system did not simply leverage the research system, but also affected it. In several ways, public involvement was a unifying force within such systems, offering a vision to unite varied actors, and supporting shared or coordinated practices. However, there were also tensions in these efforts, arising from challenges in network building in the face of extant involvement initiatives and networks.

The aspiration to embed public involvement throughout the health research enterprise was seen to offer a shared vision that could link health research organisations together:

“*I think visions are important because it unites people across the system so we are all working to the same ends through the kind of strategic processes that you put in place to make it happen.*” (E5)

Similarly, several informants highlighted the growth of a shared vision for public involvement across Alberta. Indeed, the public involvement vision might serve as a countervailing force in face of the competitive pressures that threaten the coherence and collaborative working of the health research system, as one informant explained:

“*Given the nature of research in this country, when people are competing for resources and funding, I just wonder whether* [it] *affects them, their ability to act as a system? And again, you know, you notice a lot around PPI* [patient and public involvement] *is that everything we do around PPI is intended to be collaborative… So when you encounter competitive cultures, it becomes quite difficult I think. So if there’s a question maybe to pose in the relation to this research is if you’ve got different parts of the system competing for scarce resources, what does that do in terms of systems thinking and systems leadership and systems building?*” (E6)

In addition to shared visions, commitments to public involvement could support collaborative or coordinative efforts across health research systems. Informants we spoke with were broadly supportive of efforts to share and collaborate to advance public involvement in health research. With the aim, “*at least to learn from one another*” (A2), informants identified several ways to share or coordinate, such as sharing practical ‘how to’ guides, evaluation frameworks or important standards such as those related to the reimbursement of publics who engaged in health research. For several informants, efforts to collaborate in public involvement could extend beyond sharing and involve more active coordination efforts across organisations. Speaking of efforts in Alberta in light of initiatives like the SPOR, one informant noted that efforts to collaborate were accelerating:

“*… So I think we’re moving into a much more active mode, so that’s a major recognition by the provincial health research organisation that there’s need for system level coordination and discussion …*” (A2)

Yet, there were also complexities in building collaborative networks for public involvement, given that such initiatives took place within extant systems, whose network configurations did not necessarily correspond. As one informant from England put it, building new networks “*in systems that are already in place is quite difficult*”*:*

“*I think I hesitate because the reality of trying to bring together disparate groups of people, disparate groups of organisations around a task has been quite challenging. I don’t think we would set it up in the way we set it up now, you know, you would think more carefully about what’s required to do that. So yes it would make more sense. The reality of making that happen in systems that are already in place is quite difficult. You can try it … But I think I’m just, I’m just being realistic…*” (E6)

Similarly, while supporting the principle of collaborative working, an informant from Alberta highlighted the challenge of building new public involvement initiatives where “*many of these things already existed.*” Suggesting that there were many options for advancing harmonisation that new initiatives like SPOR could promote, including ignoring extant networks, duplicating extant networks, or seeking to advise and navigate around existing organisations and networks, this informant emphasised the need for the latter approach:

“*You know, I actually think that that’s a big, big task and mandate for the SPORs and SPOR platforms, to you know harmonise, ‘cause, it’s not like there was nothing before SPOR existed... Many of these things already existed, so SPOR comes along and, you know, you view it as a multiple-choice question of how should SPOR enhance things? Well, one is, SPOR could ignore what’s already there and just recreate. Or … SPOR could try to become an entity alongside existing entities... the mandate of SPOR could be to be like a meta, a meta entity that just draws a circle around all of the existing assets and creates a little bit of a navigation map so that they’re all integrated …*. *Like I think that’s ultimately what the SPOR mandate could, should be, is to harmonise and integrate these entities.*” (A12)

Adding to the complexity of working with extant networks, new and old initiatives might have incompatible configurations by, for example, drawing different jurisdictional borders. Noting that “*a lot of the groups here actually don’t want to utilise SPOR because they’ve got their own networks and resources and infrastructure in place to support their needs*”, one informant from Alberta suggested that there was sometimes resistance to starting ‘from scratch’ and that national configurations could be misaligned with Alberta’s provincial public involvement initiatives:

“*So you can’t really re-establish, if you’ve already got a network happening already. And some of them are national, so for example, the primary care network. They have their own infrastructure and their own access to act in community groups and engage and so on … so to start from scratch, not likely.*” (A5)

#### Implications of public involvement for health systems

The public involvement initiatives we examined had arisen to advance health research that could support and improve healthcare services and systems. Informants saw considerable value in the connection between health research systems and healthcare systems and identified ways to strengthen that connection through more integrated approaches to public involvement. Few informants identified a role for the health research system beyond healthcare or reflected on the implications of this wider remit for public involvement.

The public involvement initiatives we examined had emerged through research initiatives that were focused on health services, whether through the NHS Research and Development programme and the creation of the NIHR in England, or the SPOR in Alberta. Such initiatives were aimed at the application of health research in healthcare. As one informant put it, “*We are trying to get the health delivery system connected with the health research system*” (A6). Thus, in speaking about who should be engaged, informants were principally concerned with service users, namely patients, families and carers. Involvement with these publics aimed to ensure value and address needs:

“*It ensures we get the best value and the best outcomes from the research…*” (E5)

“*So I would say the vision really is how do we take these public and patient needs into the way we are conducting and using research.*” (A14)

The relevance of public involvement in health research for healthcare led several informants to draw lessons from healthcare when reflecting on health research. These reflections sometimes pointed to areas of potential improvement in public involvement activities, in both domains. For one informant, for example, health research offered an example of a well-organised public involvement system by comparison with healthcare. For another, healthcare offered lessons on how to involve communities in health research:

“*You know, we have a high population of refugees and so on and there’s been some work to engage with these groups of people but that’s about health services, you know, and the planning and delivery of those services … and I just feel as though, you know, at some stage it must then evolve into engaging them in research.*” (E2)

Taking the opportunity for lesson-learning one step further, several informants highlighted the potential for ‘cross-fertilisation’ or ‘connections’ between the public involvement efforts within both systems. Highlighting the fact that similar people were recruited to, or affected by, involvement efforts, these informants called for “*some greater connection across service delivery and research*” (E10). As another informant explained:

“*What I have always found is that you go to one meeting that involves members of the public in research and then I’ll go to another meeting that involves members of the public in planning or delivery or you know, decisions about health services and they always tend to be the same people… So I just wonder if there is scope for thinking about public involvement of, you know, not just in research but some cross-fertilisation of other areas?*” (E2)

The contribution of public involvement to the link between health research and healthcare led one informant to suggest that health research should be understood as a component of healthcare, with all public involvement premised on that expectation:

“*What I would love is if people, members of the general public, had a sense that research was a part of healthcare and that, that there was a sort of general understanding and, and buy-in to the idea that, that research brings us better treatments, it brings us better services and participating in it and being interested in the results and helping people to decide what it is we...research is...part of your health and wellbeing.*” (E3)

Yet, though muted, a few informants pointed to a role for health research beyond healthcare. Highlighting that the health research system was comprised of several ‘sectors’, one informant noted that the vision for public involvement was not shared across them all. More pointedly, one informant expressed concern at the relative inattention to publics other than service users in health research, with implications for the types of broader community involvement often emphasised in public health research:

“*In health, particularly... health services research, which is funded by NIHR, public is defined to include … patients, carers, families … I think that 90% of the involvement in NIHR projects is actually patients.*” (E4)

## Discussion

To inform efforts in Ontario, Canada, to mobilise public involvement across the provincial health research enterprise, we conducted an exploratory qualitative descriptive study of organisational efforts to advance public involvement in health research within two jurisdictions, namely England in the United Kingdom and Alberta in Canada, which house active efforts to mobilise health research and support jurisdiction-wide public involvement efforts. In doing so, we drew on research that highlights the organisational and network relations that condition the capacity for the public involvement agenda to be advanced and on prior work by members of our team that drew on an established framework for the operation of health research systems to characterise the governance functions related to public involvement, and which identifies the essential and complex role of the stewardship function [15].

In analysing the data, we collected from expert informants across these two jurisdictions, we aimed to identify consistent themes regarding processes and challenges rather than to characterise and contrast each system. Despite marked differences between these jurisdictions – in size, research structures and histories of public involvement – we identified similar patterns and challenges in the governance of public involvement within each.

In both jurisdictions, we identified a similar set of basic building blocks that enabled public involvement to be embedded within and across health research organisations. This comprised, first, a vision of a network of connected health research actors within the jurisdiction – in other words a self-conscious ‘system’ – within which public involvement could be embedded. Informants identified with the concept of a health research system and saw its relevance to their efforts. Though not fully convinced that it operated as an effective system should, informants from both England and Alberta identified a history of efforts to build a system that linked together individuals and organisations, expectations and practices.

Against this backdrop, informants identified several strategies that supported the mobilisation of public involvement within and across systems. In both jurisdictions, strategic leadership to advance a vision of involvement was seen as an essential ingredient. However, leadership did not work through vision alone [15]. Whether advanced through research requirements, through financial incentives or through policy guidance, policy levers were mobilised to embed public involvement as a tangible activity within research organisations and the research enterprise. In doing so, these tangible efforts provided organisational infrastructure as well as human and financial resources to enable public involvement initiatives to be put into practice.

The mobilisation of these basic building blocks was seen to have enabled each jurisdiction to go some way toward embedding public involvement in health research. There was, as we report, a considerable sense of satisfaction with respect to achievements to date – sufficient to encourage the supporters and stewards of public involvement to seek to enrich and deepen sustained effort, and to address emerging and longstanding challenges in the processes and impacts of public involvement.

Reflecting on the culture change that had begun to normalise public involvement in health research, informants suggested that such efforts were now at a ‘crossroads’, with the potential to improve public involvement processes. This involved, first, an intent to move beyond the initial and simple aim that publics just ‘be involved’, to more purposeful practice, perhaps reflecting broad concern with issues of ‘tokenism’ [7, 10, 42]. Additionally, improvements were needed to address the challenge of diversity in public involvement. Raised by informants as an area of emerging awareness and concern, this issue will require attention at the systems level, including organised effort and perhaps structural change [43].

In addition, we identified two areas of challenge for realising and assessing the impacts of public involvement activity. The first was the issue of research priority-setting, which is known to be a “*value-laden and political*” process [43] that may provoke resistance for seeming to threaten the traditional reliance on the priorities of scientists [44], as well as “*established research structures, procedures and cultures*” [5]. This issue was one that we intentionally probed, and approached with prior awareness of the differences between the two jurisdictions in established processes. Yet, while these differences were seen, common problems remained, with persistent ‘mismatches’ in priorities between researchers and publics, limitations in follow-through where public priorities were identified, and differentials in capacity and authority affecting their uptake.

The second issue concerned the question of success itself and, specifically, how the success or impact of public involvement could or should be evaluated. This is an area of active effort in many jurisdictions [8, 12–14, 45, 46], but also raises philosophical, epistemological and methodological concerns [1, 6, 9, 16, 47]. Aligned with the distinction between formative and summative evaluation, informants conceived of different purposes for evaluation, which might improve the enterprise of public involvement, but also – or instead – appraise its value. This latter approach to evaluation was explicitly challenged by some informants, who questioned both the feasibility and appropriateness of subjecting public involvement to such a test.

Finally, we identified ways in which efforts to embed public involvement as a core component of health research do not simply mobilise the capacity of health research systems, but affect them. This transpires, first, because of the network building dimensions of the public involvement agenda, which have the potential to strengthen research systems through shared visions and collaborative efforts. At the same time, research systems comprise a multiplicity of not-always-congruent networks, and tensions between these networks can encourage dissolution rather than integration. Thus, care and caution are needed to leverage extant public involvement capacity, rather than ignore or duplicate it, as health research systems work to spread and scale public involvement.

A second way in which public involvement affects the health research system arises from its orientation toward the health system. In the health research systems we examined, research was clearly orientated to the healthcare components of health systems, with the associated emphasis on publics as service users and system advisors (i.e. publics as patients, families, caregivers). Informants identified important ways in which public involvement strengthened the health research system’s capacity to serve the healthcare system, and also ways in which public involvement in both systems could be strengthened through knowledge exchange or coordination across systems. However, there was limited attention to the health research and publics needed to attend to other dimensions of health systems such as public health, social care, health-in-all policy and related issues.^1^ Whether this is a systemic problem within these research systems or an artefact of our research approach is hard to know. We would argue that the orientation to healthcare identified here fairly reflects the actual orientation of public sector agencies familiar with the public involvement and public engagement discourse in each jurisdiction, and thus does reflect a limited vision of the scope and capacity of public involvement in health research with respect to health systems. That said, research systems are comprised of multiple, not necessarily linked, networks, and we cannot speak to the presence or absence of robust public involvement infrastructure in each jurisdiction focused on research related to community-based or social care, public health or health-in-all policy, and capable of supporting improvements in those critical health system subsectors.

The limitations of this exploratory study are several. We examined only two jurisdictions, which vary markedly, but did not comprehensively compare and contrast these as distinct cases. Thus, while we identified similar processes and challenges in the governance of public involvement across jurisdictions, we did not explore the political contexts that underpin the formation and evolution of public involvement activity. Additionally, we learned about these jurisdictions through interviews with a limited number of key informants and were not successful in engaging informants in equal numbers across jurisdiction, or by type of informant. The informants who agreed to speak with us were likely committed to the public involvement agenda, thus providing a more positive interpretation of efforts and accomplishments than might have been provided by other members of their organisations or by those who did not agree to participate. Finally, the informants who spoke with us emphasised public involvement in clinical and health services research. Because the health research system framework orients to health systems – a concept that includes but extends beyond healthcare to encompass public health and other policy efforts aimed at supporting and sustaining health – we sought out informants from organisations with a public health orientation in both jurisdictions, anticipating also that this might illuminate issues related to the involvement of communities. However, we lacked the time and resources to pursue a more sustained exploration of the networks of organisations in public health, community-based care or social care, which may not be linked to the organisations we engaged, or utilise the public involvement discourse that is prevalent within healthcare policy circles. Additionally, although we sought informants from health research funding agencies with a broad health research mandate (e.g. CIHR, United Kingdom Medical Research Council, Alberta Innovates), we made no additional effort to correct for the relatively poor representation of public involvement in basic biomedical research within our sample.

## Conclusion

This study offers important insight for governance for public involvement in health research, highlighting the role of research organisations, and the essential function of system stewardship. Further, it points to the two dimensions of governance that public involvement implicates [15]. One dimension of governance for public involvement relates to its role within health research systems, raising questions about how system stewards can embed and enable public involvement activity. Here, we suggest that a reflexively self-aware health research system may contribute to the advancement of public involvement, providing a supportive vision of collaborative action as well as a set of networked actors. Further, public involvement initiatives may have the potential to reinforce the ties that bind system actors, especially where care is taken to leverage rather than sideline extant networks. Public involvement may also reinforce the orientation of health research toward health systems. In particular, from the perspective of publics as service users and system planners, there may be potential to better coordinate involvement between health research and healthcare systems, sharing capacity and good practice. There is, however, also a risk that public involvement will reinforce tendencies to orient health research almost exclusively toward healthcare, and sideline population and public health, including through public health or health-in-all research. As the public involvement movement strengthens and deepens across the health research enterprise, it will be important to ensure that public involvement is also used to support health research systems that address the needs of health systems beyond healthcare.

The second dimension of governance for public involvement relates to its role for health research systems, raising questions about the role of publics as governors of such systems. While not the focus of this study, these issues were illuminated in discussions about areas for improvement and of persistent challenge, which highlighted tensions over the ultimate aim of public involvement in health research, and between a consumerist or more emancipatory vision. Whether public involvement should be used to galvanise public interest in and support for existing arrangements [20], or serve as a force to challenge stabilised regimes that privilege scientists [2] and subject scientific practice to social judgment [27], are not questions that are amenable to easy resolution. However, active efforts to govern for public involvement in research systems might help to front such concerns and debates, and foster a role for publics as both partners within, and arbiters of, health research systems [15].

## Abbreviations

CIHR: Canadian Institutes of Health Research
NHS (United Kingdom): National Health Service
NIHR (United Kingdom): National Institute for Health Research,
SPOR (Canada): Strategy for Patient Oriented Research

## Footnotes

1 According to Pang et al. [26], health research systems exist to serve health systems, including healthcare systems, public health systems and cognate systems that are directly aimed at protecting or promoting health (e.g. seat belt policy for transportation).

## References

[1] Staley K, Buckland SA, Hayes H, Tarpey M (2014). ‘The missing links’: understanding how context and mechanism influence the impact of public involvement in research. Health Expect.

[2] Caron-Flinterman JF, Broerse JE, Bunders JF (2007). Patient partnership in decision-making on biomedical research: changing the network. Sci Technol Human Values.

[3] Israel BA, Schulz AJ, Parker EA, Becker AB (2001). Community-based participatory research: policy recommendations for promoting a partnership approach in health research. Educ Health (Abingdon)..

[4] Ahmed SM, Beck B, Maurana CA, Newton G (2004). Overcoming barriers to effective community-based participatory research in US medical schools. Educ Health (Abingdon).

[5] Caron-Flinterman JF, Broerse JE, Bunders JF (2005). The experiential knowledge of patients: a new resource for biomedical research?. Soc Sci Med.

[6] Boote J, Telford R, Cooper C (2002). Consumer involvement in health research: a review and research agenda. Health Policy.

[7] Domecq JP, Prutsky G, Elraiyah T, Wang Z, Nabhan M, Shippee N (2014). Patient engagement in research: a systematic review. BMC Health Serv Res.

[8] Staley K (2009). Exploring Impact: Public Involvement in NHS, Public Health and Social Care Research..

[9] Staley K (2015). ‘Is it worth doing?’ Measuring the impact of patient and public involvement in research. Res Involv Engagem.

[10] Brett J, Staniszewska S, Mockford C, Herron-Marx S, Hughes J, Tysall C (2014). A systematic review of the impact of patient and public involvement on service users, researchers and communities. Patient.

[11] Berta W, Virani T, Bajnok I, Edwards N, Rowan M (2014). Understanding whole systems change in health care: insights into system level diffusion from nursing service delivery innovations–a multiple case study. Evid Policy.

[12] Staniszewska S, Brett J, Mockford C, Barber R (2011). The GRIPP checklist: strengthening the quality of patient and public involvement reporting in research. Int J Technol Assess Health Care.

[13] Sophie S, Ade A, Rosemary B, Peter B, Louca-Mai B, Jo B (2011). Developing the evidence base of patient and public involvement in health and social care research: the case for measuring impact. Int J Consum Stud.

[14] Brett J, Staniszewska S, Simera I, Seers K, Mockford C, Goodlad S (2017). Reaching consensus on reporting patient and public involvement (PPI) in research: methods and lessons learned from the development of reporting guidelines. BMJ Open.

[15] Miller FAPS, Dobrow MJ, Berta W (2018). Public involvement in health research systems: a governance framework. Health Res Policy Syst.

[16] Evans D (2014). Patient and public involvement in research in the English NHS: a documentary analysis of the complex interplay of evidence and policy. Evid Policy..

[17] Saunders C, Girgis A (2010). Status, challenges and facilitators of consumer involvement in Australian health and medical research. Health Res Policy Syst.

[18] O’Donnell M, Entwistle V (2004). Consumer involvement in research projects: the activities of research funders. Health Policy.

[19] Van Bekkum JE, Hilton S (2014). UK research funding bodies’ views towards public participation in health-related research decisions: an exploratory study. BMC Health Serv Res.

[20] Van Bekkum JE, Fergie GM, Hilton S (2016). Health and medical research funding agencies’ promotion of public engagement within research: a qualitative interview study exploring the United Kingdom context. Health Res Policy Syst..

[21] Hanney S, Kuruvilla S, Soper B, Mays N (2010). Who needs what from a national health research system: lessons from reforms to the English Department of Health's R&D system. Health Res Policy Syst..

[22] Hanney SR, González-Block MA (2013). Organising health research systems as a key to improving health: the world health report 2013 and how to make further progress. Health Res Policy Syst..

[23] Green G (2016). Power to the people: to what extent has public involvement in applied health research achieved this?. Res Involv Engagem.

[24] Dodgson R Lee K, Drager N. Global Health Governance: A Conceptual Review. Centre on Global Change and Health, London School of Hygiene & Tropical Medicine/World Health Organization. https://researchonline.lshtm.ac.uk/18031/1/a85727_eng.pdf. Accessed 24 Aug 2018.

[25] Saltman RB, Ferroussier-Davis O (2000). The concept of stewardship in health policy. Bull World Health Organ.

[26] Pang T, Sadana R, Hanney S, Bhutta ZA, Hyder AA, Simon J (2003). Knowledge for better health: a conceptual framework and foundation for health research systems. Bull World Health Organ.

[27] Milewa T, Buxton M, Hanney S (2008). Lay involvement in the public funding of medical research: expertise and counter-expertise in empirical and analytical perspective. Crit Public Health.

[28] INVOLVE. What is Public Involvement in Research? 2017. http://www.invo.org.uk/find-out-more/what-is-public-involvement-in-research-2/. Accessed 24 Aug 2018.

[29] Canadian Institutes of Health Research. Patient Engagement. 2014. http://www.cihr-irsc.gc.ca/e/45851.html. Accessed 24 Aug 2018.

[30] Kvale S, Brinkmann S (2009). Interviews: Learning the Craft of Qualitative Research Interviewing.

[31] Charmaz K (2006). Constructing Grounded Theory: A Practical Guide Through Qualitative Analysis..

[32] Glaser BG, Strauss AL (2009). The Discovery of Grounded Theory: Strategies for Qualitative Research.

[33] Thorne S (2000). Data analysis in qualitative research. Evid Based Nurs.

[34] Office for National Statistics. Population Estimates for UK, England and Wales, Scotland and Norther Ireland: mid-2016. 2016. https://www.ons.gov.uk/peoplepopulationandcommunity/populationandmigration/populationestimates/bulletins/annualmidyearpopulationestimates/mid2016. Accessed 24 Aug 2018.

[35] Statistics Canada. Population by Year, by Province and Territory 2016. https://www150.statcan.gc.ca/t1/tbl1/en/tv.action?pid=1710000501. Accessed 24 Aug 2018.

[36] United Kingdom Department of Health. Best Research for Best Health: A New National Health Research Strategy. 2005. https://www.gov.uk/government/uploads/system/uploads/attachment_data/file/568772/dh_4127152_v2.pdf. Accessed 24 Aug 2018.

[37] National Institute for Health Research. Going the Extra Mile: Improving the Nation’s Health and Wellbeing through Public Involvement in Research. 2015. https://www.nihr.ac.uk/patients-and-public/documents/Going-the-Extra-Mile.pdf. ​Accessed 24 Aug 2018.

[38] Canadian Institutes of Health Research. Strategy for Patient-Oriented Research (SPOR). 2011. http://www.cihr-irsc.gc.ca/e/documents/P-O_Research_Strategy-eng.pdf. Accessed 24 Aug 2018.

[39] Canadian Institutes of Health Research. Strategy for Patient-Oriented Research (SPOR) Patient Engagement Framework. 2014. http://www.cihr-irsc.gc.ca/e/documents/spor_framework-en.pdf. Accessed 24 Aug 2018.

[40] Alberta Health Services. Patient First Strategy 2015. http://www.albertahealthservices.ca/assets/info/pf/first/if-pf-1-pf-strategy.pdf. Accessed 24 Aug 2018.

[41] Shklarov S, Marshall DA, Wasylak T, Marlett NJ. “Part of the Team”: Mapping the outcomes of training patients for new roles in health research and planning. Health Expect. 2017;20(6):1428–36. 10.1111/hex.12591 PMC5689226 28660732

[42] Shippee ND, Domecq Garces JP, Prutsky Lopez GJ, Wang Z, Elraiyah TA, Nabhan M (2015). Patient and service user engagement in research: a systematic review and synthesized framework. Health Expect.

[43] Pratt B, Merritt M, Hyder AA (2016). Towards deep inclusion for equity-oriented health research priority-setting: a working model. Soc Sci Med.

[44] Petit-Zeman S, Firkins L, Scadding JW (2010). The James Lind Alliance: tackling research mismatches. Lancet.

[45] Popay J, Collins M, with the PiiAF Study Group. The Public Involvement Impact Assessment Framework Guidance. 2014. http://piiaf.org.uk/documents/piiaf-guidance-jan14.pdf. Accessed 24 Aug 2018.

[46] Abelson J, Li K, Wilson G, Shields K, Schneider C, Boesveld S (2016). Supporting quality public and patient engagement in health system organizations: development and usability testing of the public and patient engagement evaluation tool. Health Expect.

[47] Abelson J, Gauvin F-P (2006). Assessing the Impacts of Public Participation: Concepts, Evidence and Policy Implications.

